# Mating Patterns and Bt Resistance: The Case of the European Corn Borer

**DOI:** 10.1371/journal.pbio.0040214

**Published:** 2006-05-30

**Authors:** Liza Gross

The main argument for planting transgenic, pest-resistant crops rests on their potential to reduce widespread pesticide spraying. Such benefits require that agricultural land-use strategies impede the evolution of pest resistance. But such strategies—including the high-dose refuge (HDR) strategy mandated in the United States—are based on key assumptions about pest biology. Insect fitness depends on finding the optimal balance between when to mate and when to set off for greener pastures—mating before dispersal increases the risk of inbreeding, but dispersing before mating increases the risk of never finding a mate. Pest management depends on understanding and using this balance to prevent the evolution of insect resistance.

As important as mating and dispersal are to thwarting resistance, empirical evidence on these behaviors in major crop pests—including the European corn borer *(Ostrinia nubilalis)*, targeted by *Bacillus thuriengiensis (Bt)* toxins produced by transgenic insecticidal corn—is limited. While European corn borers *can* disperse over half a mile—the maximum distance allowed between a *Bt* field and the corresponding refuge—and even up to 20 miles, it's unclear whether they do so routinely, take flight before or after mating, or show any preference for resident or immigrant mates.

To gain a better understanding of the European corn borer's mating and dispersal habits, Ambroise Dalecky, Sergine Ponsard, Richard Bailey, Céline Pélissier, and Denis Bourguet released and recaptured moths in French crop fields over three breeding seasons in 2004 and 2005. They found that females mate randomly with resident and immigrant males—an important HDR assumption, since random mating dilutes resistance genes. But they also discovered that some males and females mate before dispersing—suggesting a possibly more restricted mating range than previously assumed.

The HDR strategy assumes that a patchwork of *Bt* cornfields interspersed with untreated refuges will facilitate gene flow between the areas. Resistance gene variants, or alleles, are found in either one (heterozygotes) or two (homozygotes) copies in individuals who carry them. Only homozygotes are resistant to (that is, survive the *Bt* toxins), while heterozygotes die as larvae if they feed on such plants. HDR management aims to eliminate as many resistance alleles from each generation as possible by maximizing the proportion of heterozygotes (where resistance alleles are available for purging) and maximizing the proportion of these heterozygotes developing in *Bt* fields (where resistance alleles are indeed purged), rather than in refuges. If substantial mating takes place before dispersal, there will be more homozygotes, which means fewer purging opportunities—and a potentially less effective HDR strategy. If crops are not rotated and if post-mating dispersal is low, this deleterious effect of low pre-mating dispersal can be offset by having a higher proportion of heterozygous eggs laid on *Bt* corn plants and dying at larval stage. In other cases, however, low pre-mating dispersal is predicted to reduce the efficacy of the HDR strategy.

To track moth mating and dispersal habits, the researchers relied on biochemical markers—consisting of different carbon signatures produced by wheat and corn, which are in turn evident in lab-raised, wheat-fed moths, and corn-fed field moths—and color-coded ink spots applied to adults before release. Male and female pupae were separated to prevent mating before they were released less than 24 hours after they emerged from their pupal case.

In 17 sessions over three weeks, nearly 8,800 virgin moths were released during the June 2004 breeding season into four different cornfields. Soybean and wheat—crops that aren't targeted by corn borers—grew in the fields the year before, so no pupae were present as if the field had been planted with *Bt* corn. Refuge facsimiles were provided by adjacent fields planted with corn in 2003, the most likely source of wild moths at the 2004 study sites.

Thirty-six hours after release, marked moths and unmarked moths were captured along the field borders, where the moths typically mate and rest. The proportion of marked individuals that were recaptured varied significantly between sites and sessions but not between sexes, averaging about 4%. About 97% of recaptured females had mated—indiscriminately with resident and immigrant males, the researchers determined, by analyzing the carbon content of the sperm packets, or spermatophores, they acquired during mating. Resident males rarely mated with immigrant females, perhaps because immigrant females tended to arrive already mated.

Because wild moths might disperse sooner than 24 hours, the researchers placed corn borer pupae directly in a cornfield the following season, then watched and waited. Moths first took wing about 3.5 to 9.5 hours after emerging, and their flight movements were unlikely to take them farther than 220 feet within the first 24 hours—indicating that high rates and high distances of dispersal were unlikely to occur before mating and that far more than 4% were still present and hence likely to mate within their natal range.

The researchers go on to confirm that a “substantial proportion”—up to about 57% for females—of newly emerged corn borers mate at a very local scale before dispersing. These findings suggest that the HDR strategy may not ensure substantial gene flow between susceptible and resistant moths. This disconnect between the evidence and the HDR model indicates that other assumptions—including whether females who mate close to home also lay their eggs there—should be rigorously tested before counting on HDR to circumvent the resistance machinery of its targets.

## 

**Figure:**
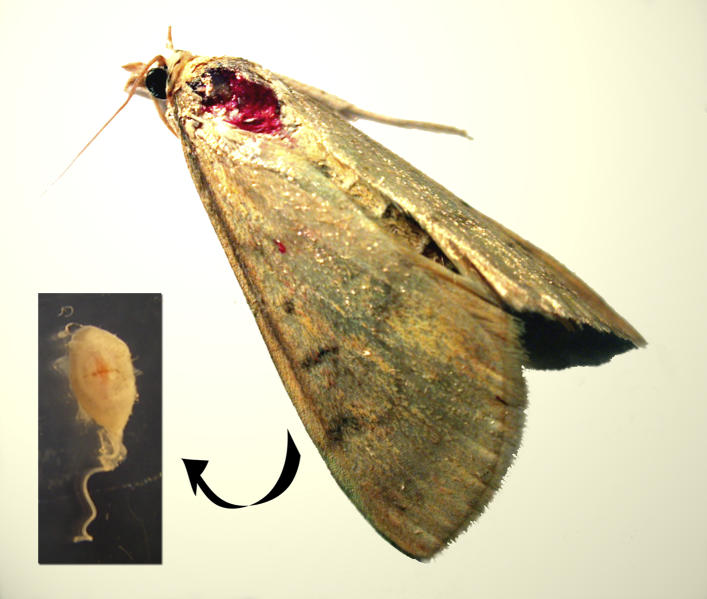
Dispersal and mating patterns in European corn borer moths: a color-marked female and a biogeochemically markedspermatophore (shown at about the same scale). (Image: Dalecky et al., Laurent Pélozuelo, and Laurent Soldati)

